# Association of right precuneus compression with apathy in idiopathic normal pressure hydrocephalus: a pilot study

**DOI:** 10.1038/s41598-022-23800-x

**Published:** 2022-11-28

**Authors:** Yoshihiro Chadani, Tetsuo Kashibayashi, Takahiro Yamamoto, Atsushi Tsuda, Ryoko Fujito, Masanori Akamatsu, Naoto Kamimura, Ryuichi Takahashi, Takuji Yamagami, Hirokazu Furuya, Tetsuya Ueba, Motoaki Saito, Keiji Inoue, Hiroaki Kazui

**Affiliations:** 1 Department of Neuropsychiatry, Kochi Medical School, Kochi University, Kohasu Oko-Cho, Nankoku, Kochi 783-8505 Japan; 2 Department of Psychiatry, Hyogo Prefectural Harima-Himeji General Medical Center, Himeji, Hyogo Japan; 3 Department of Rehabilitation, Kochi Medical School, Kochi University, Kohasu Oko-Cho, Nankoku, Kochi Japan; 4 Health Service Center Medical School Branch, Kochi University, Kohasu Oko-Cho, Nankoku, Kochi Japan; 5 Dementia-Related Disease Medical Center, Departments of Neurology and Cognitive Disorders, Hyogo Prefectural Rehabilitation Hospital at Nishi-Harima, Tatsuno, Hyogo Japan; 6 Department of Diagnostic and Interventional Radiology, Kochi Medical School, Kochi University, Kohasu Oko-Cho, Nankoku, Kochi Japan; 7 Department of Neurology, Kochi Medical School, Kochi University, Kohasu Oko-Cho, Nankoku, Kochi Japan; 8 Department of Neurosurgery, Kochi Medical School, Kochi University, Kohasu Oko-Cho, Nankoku, Kochi Japan; 9 Department of Pharmacology, Kochi Medical School, Kochi University, Kohasu Oko-Cho, Nankoku, Kochi Japan; 10 Department of Urology, Kochi Medical School, Kochi University, Kohasu Oko-Cho, Nankoku, Kochi Japan

**Keywords:** Neuroscience, Psychology, Medical research

## Abstract

Apathy is frequently observed in idiopathic normal pressure hydrocephalus (iNPH) and worsens cognitive impairment and gait disturbance. In this study, we evaluated the regions associated with apathy in iNPH using statistical imaging analysis on the whole brain, both in terms of cerebral blood flow and gray matter volume. Twenty-seven patients with iNPH were assigned to two groups based on their scores on the neuropsychiatric inventory items related to apathy; 18 patients were assigned to the group with apathy (iNPH + APA) and 9 to the group without apathy (iNPH − APA). The magnetic resonance images and cerebral blood flow single-photon emission computed tomography data of the two groups were compared using statistical parametric mapping 12. The regional gray matter volume of the right precuneus was significantly larger in the iNPH + APA group than in the iNPH − APA group, but the regional cerebral blood flow in any region of the brain was not significantly different between the two groups. These results suggested that the larger gray matter volume, which is thought to reflect gray matter compression, in the precuneus might be involved in apathy in iNPH.

## Introduction

Idiopathic normal pressure hydrocephalus (iNPH) is a slowly progressive syndrome caused by excessive accumulation of cerebrospinal fluid (CSF) and is typically characterized by a triad of symptoms, including gait, cognitive, and urinary disturbances, and ventricular dilation^1,2^. In addition to this triad of symptoms, apathy is frequently observed in iNPH^3,4^. Apathy has been generally known to worsen cognitive impairment and cause impaired activities of daily living (IADL) in patients with Alzheimer’s disease (AD)^5,6^ and Parkinson’s disease (PD)^7^. Similarly, gait disturbance and cognitive impairment in iNPH are more severe in patients with apathy than in those without apathy^8,9^. Therefore, apathy is an important symptom that can exacerbate cognitive and motor impairment, as well as IADL, regardless of the cause. However, the neurological basis of apathy that develops in iNPH is unclear.

The most distinctive morphologic changes to the brain in iNPH are enlargement of the ventricles and Sylvian fissures and tightness of the high-convexity and medial subarachnoid spaces^10^. These features were named disproportionately enlarged subarachnoid space hydrocephalus (DESH)^11^ and are also emphasized in the Japanese guidelines for the management of iNPH^2^. In addition to the DESH findings, a relatively high density of gray matter in the high-convexity areas, particularly the precuneus, was reported in patients with iNPH using voxel-based morphometry (VBM), a method of statistical imaging analysis^12^. Furthermore, this increase in gray matter density in the high-convexity areas on VBM was considered to share the same phenomenon with the tightness of sulci; therefore, an increase in gray matter density reflects compression of the brain in iNPH.

In a study on the relationship between the brain morphologic changes and clinical symptoms in iNPH, it was shown that a larger Evans index, which indicates the degree of enlargement of the lateral ventricles, measured on head computed tomography images was associated with severe urinary disturbance, sharper angle of the corpus callosum with cognitive impairment and motor disturbance, and ventricle width with the severity of all of the triad symptoms^13^. Moreover, it was found that severe thinning of the dorsal corpus callosum on magnetic resonance imaging (MRI) was associated with the severity of gait, cognitive, and urinary disturbance^14^. It was also reported that ventricular enlargement correlated with severe apathy in iNPH^15^. However, there had been no studies on the relationship between apathy and regional gray matter volume (rGMV) using VBM in a whole-brain analysis.

Cerebral blood flow (CBF) single-photon emission computed tomography (SPECT) studies have shown that anterior-dominant CBF reduction was the most common CBF alteration in iNPH, but mixed or diffuse and posterior-dominant CBF reduction have been shown to exist^16^. Although studies on the relationship between decreased CBF and the triad of symptoms in iNPH are abundant, their outcomes have not been consistent^17–23^. It was reported that apathy was associated with reduced CBF in the right caudate nucleus^24^. However, that study used the region of interest (ROI) method, and to the best of our knowledge, no studies have investigated the whole-brain to determine the relationship between CBF and apathy.

Apathy in iNPH may be associated with changes in brain morphology and CBF. Therefore, this study aimed to identify the brain regions associated with apathy in iNPH by analyzing both regional gray matter volume (rGMV) and regional CBF (rCBF) data with whole-brain analysis using statistical parametric mapping.

## Methods

### Flow of care for iNPH at Kochi University Medical Hospital

At Kochi University Medical Hospital, specialists in geriatric psychiatry, neurology, neuro-urology, and neuroradiology attend to the clinical and neuroimaging examinations of patients who meet the diagnostic criteria for possible iNPH, and neurosurgeons perform shunt surgery on patients deemed eligible. The diagnostic criteria and treatment procedures for patients with iNPH followed the Japanese clinical guidelines for iNPH second edition^25^.

Preshunt surgery examinations, including CSF tap test were primarily performed in the psychiatry department.

Cognition was assessed with the following tests: mini-mental state examination (MMSE)^26^, frontal assessment battery (FAB)^27^, digit symbol substitution test (DSST) of the Wechsler adult intelligence scale-III, a subtest of immediate and delayed recall of a short story of the Rivermead behavioral memory test (RBMT)^28^, and the Japanese version of the Addenbrooke’s cognitive examination 3 (ACE-3)^29,30^. The everyday memory checklist (EMC)^31^ was used to assess amnesia of the patient in daily life for the patient and caregivers.

Gait speed was assessed with the timed up and go test (TUG)^32^ and 10-m reciprocating walking test (WT). In addition, the quality of gait was assessed using the gait status scale-revised (GSSR)^33^, which examines 10 factors of gait disturbances: (1) postural stability (range, 0–4), (2) independence of walking (0–2), (3) wide base gait (0–1), (4) lateral sway (0–2), (5) petit pas gait (0–2), (6) festinating gait (0–2), (7) freezing of gait (0–2), (8) disturbed tandem walking (0–1), (9) shuffle (0–1), and (10) bow-leggedness (0–1). We used the total score of the 10 items of the GSSR to obtain scores that ranged from 0 to 18.

The urinary disturbance was assessed with the Overactive bladder symptom score (OABSS)^34^ and the international prostate symptom score (IPSS)^35^.

The triad of symptoms was evaluated with the iNPH grading scale (iNPHGS)^33^, a clinician-rated scale that separately evaluated the severity of each of the symptoms of the triad on a scale of 0 to 4. The Japanese version of the modified Rankin scale (mRS)^36^ assessed the level of independence in ADL, with possible scores of 0–6. The overall severity of dementia was rated on a 5-point scale (i.e., 0, 0.5, 1, 2, or 3) using the clinical dementia rating (CDR). The caregiver burden was assessed with the Zarit burden interview (ZBI)^37^. Higher scores indicate better performance in the DSST, RBMT story recall, MMSE, FAB, and ACE-3 and worse performance in the EMC, GSSR, OABSS, IPSS, iNPHGS, mRS, CDR, and ZBI.

Using the NPI^38^, psychiatric symptoms were assessed by asking the caregiver of the patient for the presence of 12 types of psychiatric symptoms within 30 days before the evaluation: delusions, hallucinations, agitation, dysphoria, anxiety, euphoria, apathy, disinhibition, irritability, aberrant motor behavior, nighttime behavioral disturbances, and appetite and eating abnormalities. Each symptom was graded in terms of its frequency on a scale of 1 to 4 and its severity on a scale of 1 to 3, and the total score (frequency × severity) was calculated. In addition, apathy was assessed using a self-rating Japanese version of the apathy scale (AS)^39^. Higher scores indicated worse symptoms in the NPI and AS.

A lumbar tap was performed for patients with possible iNPH. The CSF samples were transferred into a 15-mL polypropylene tube, centrifuged at 1500 rpm, and stored at − 80 °C. The phosphorylated tau level of the CSF sample was measured by enzyme-linked immunosorbent assay.

The neuroimaging tests performed on all patients were as follows: (1) head MRI (Phillips’ Ingenia 3.0 Tesla), including 3D volume T1-weighted, T2-weighted, fluid-attenuated inversion recovery, and susceptibility-weighted imaging sequences; (2) CBF SPECT, which was done 10 min after intravenous injection of 111-MBq N-isopropyl-p- [123I]-iodoamphetamine; and (3) [^123^I]-2beta-carbometoxy-3beta-(4-iodophenyl)-N-(3-fluoropropyl) nortropane (FP-CIT) (ioflupane) SPECT, which was done 3 h after intravenous injection of 167-MBq [^123^I] FP-CIT. The DaT View software (Nihon Medi-Physics, Tokyo, Japan) was used to calculate the Specific Binding Ratio (SBR)^40^.

### Selection of subjects for this study

This was a retrospective study approved by the ethics committee of the Kochi University Medical Hospital (Kochi, Japan) and followed the Guidelines for Good Clinical Practice and the Declaration of Helsinki. As the study was a retrospective observational study, in which data were collected from medical records, an opt-out approach was used; that is, information regarding our study was provided on our homepage, and each participant’s consent to take part in our study was considered to be obtained unless they expressed their desire to be excluded. The exemption on informed consent and adoption of the opt-out approach were approved by the ethics committee of Kochi University Medical Hospital.

The inclusion criteria were as follows; (1) Patients who were hospitalized in the Department of Psychiatry between March 2018 and June 2021 and (2) those who met the Japanese clinical guidelines for iNPH second edition diagnostic criteria for probable iNPH^25^. The criteria for probable iNPH were as follows: (1) age > 60 years; (2) presence of at least one of the iNPH triad of symptoms; (3) presence of ventricular enlargement on MRI (Evans index > 0.3); (4) absence of other diseases, conditions, or radiologic findings that could be causing the clinical manifestations; (5) no history or evidence of underlying disease that can cause secondary NPH; (6) normal CSF pressure (i.e., ≤ 20 cm H_2_O) and contents; and (7) one of the following: gait disturbance and DESH or improvement of symptoms after removal of 30cc of CSF via lumbar tap. Improvement of symptoms after the lumbar tap was defined as an improvement of ≥ 3 points in the MMSE or improvement of 10% or more in the TUG test or WT.

### Statistical analysis

This study classified the subjects into two groups, according to the presence (iNPH + APA group) or absence (iNPH − APA group) of apathy on NPI. The results of cognitive tests and clinical assessments of iNPH were compared between the two groups using Mann–Whitney U test or Fisher’s exact test. Statistical analyses were performed with SPSS ver.25 (IBM SPSS statistics 25), and the significance level was set at *p* < 0.05.

#### Intergroup comparison of voxel-wise rGMV

In this study, the diffeomorphic anatomical registration through exponentiated lie algebra (DARTEL) algorithm implemented in the statistical parametric mapping 12 (SPM12; Wellcome Department of Cognitive Neurology, London, UK) was used to normalize the MR images. DARTEL is a suite of tools for achieving more accurate inter-subject registration of brain images. The MR images were segmented to gray matter, white matter, and CSF spaces, according to a tissue probability map. Segmentation results were visually confirmed for each patient by Y.C. and T.K. Using the segmented gray matter images of all the subjects, a DARTEL template was created. The original MR image of each subject was transformed into stereotactic anatomical space with deformation parameters and the template that was created in the DARTEL deformation process of the MR images. Next step, they were smoothed with an 8-mm Gaussian filter. Artifacts can be introduced when transformed into the stereotactic anatomical space, these effects are masked by the smoothing. After smoothing the images with an 8-mm Gaussian filter, SPM12 were used for voxel-wise comparisons between iNPH + APA and iNPH − APA groups using a two-sample t-test. A comparison of rGMV was performed using age; sex; the iNPHGS scores for cognitive impairment, gait disturbance, and urinary disturbance; the NPI score for dysphoria; the MMSE score and intracranial volume as covariates. The initial voxel threshold was set at *p* < 0.001 uncorrected. A cluster was considered significant if it was below a cluster-corrected *p*(FWE) < 0.05. The brain regions were identified using the atlas of neuromorphometrics implemented in SPM12.

#### Intergroup comparison of voxel-wise rCBF

Each SPECT image was first co-registered with the MR image of the subject, before being transformed into stereotactic anatomical space with deformation parameters and the template that was created in the DARTEL deformation process of the MR images. After smoothing the SPECT image with a 4-mm Gaussian filter, SPM12 was used for voxel-wise comparisons between iNPH + APA and iNPH − APA groups using a two-sample t-test. The covariates in the rCBF comparison were; age; sex; the iNPHGS scores for cognitive impairment, gait disturbance, and urinary disturbance; the NPI score for dysphoria; and the MMSE score. The initial voxel threshold was set at *p* < 0.001 uncorrected. The cluster was considered significant if it was below a cluster-corrected *p*(FWE) < 0.05. The brain regions were identified using the atlas of neuromorphometrics implemented in SPM12.

### Ethical approval and consent to participate

This study was approved by the ethics review board of the Kochi University Medical Hospital, Japan (2021-85).

## Results

### Demographic data and results of clinical assessments of the iNPH patients

Of 28 consecutive patients diagnosed with probable iNPH, 27 who had available head MRI and CBF SPECT data were included. One case was excluded from the analysis due to incorrect MRI segmentation. The iNPH + APA group comprised 18 patients, whereas the iNPH – APA group had 9. The characteristics of each group are shown in Table 1. The NPI apathy score, the GSSR score, was significantly different between the two groups.

**Table 1:** Characteristics of the iNPH + APA and iNPH – APA groups.

	iNPH + APA		iNPH – APA		*p*^a^
	No.	Mean(SD)	No.	Mean (SD)	
Sex (M:F)	18	9:9	9	7:2	0.231^b^
NPI-apathy	18	4.8 ± 2.7	9	0.0 ± 0.0	< 0.001*
AS (/42)	18	22.1 ± 8.9	8	17.0 ± 9.0	0.216
Age (years)	18	77.7 ± 6.0	9	79.0 ± 6.0	0.231
MMSE (/30)	18	21.3 ± 5.1	9	22.6 ± 4.3	0.705
FAB (/18)	18	11.3 ± 2.6	9	11.6 ± 2.5	0.820
ACE-3(/100)	12	70.6 ± 12.7	6	69.0 ± 8.9	0.750
WAIS-III DSST	17	28.4 ± 11.2	9	28.7 ± 10.5	0.833
RBMT story IR (/25)	18	5.6 ± 3.1	8	6.5 ± 2.6	0.461
RBMT story DR (/25)	18	1.9 ± 3.0	8	1.7 ± 2.0	0.849
EMC (self) (/39)	17	12.0 ± 6.3	9	10.4 ± 5.5	0.751
EMC (caregiver) (/39)	16	14.8 ± 6.3	8	9.8 ± 5.3	0.093
TUG (s)	17	23.9 ± 23.8	8	14.8 ± 4.5	0.175
10-m reciprocating WT(s)	17	35.3 ± 22.7	8	23.9 ± 5.4	0.344
GSSR (/18)	17	7.4 ± 4.1	9	4.0 ± 2.2	0.039*
OABSS	13	4.6 ± 4.0	7	2.3 ± 1.6	0.351
IPSS	13	6.2 ± 5.0	7	6.4 ± 6.6	0.877
iNPHGS gait (/4)	18	2.1 ± 0.7	9	1.7 ± 0.7	0.322
iNPHGS cognition (/4)	18	2.3 ± 0.8	9	2.3 ± 0.9	0.900
iNPHGS urination (/4)	18	1.9 ± 1.2	9	1.4 ± 1.1	0.375
mRS (/6)	18	2.4 ± 0.8	9	1.8 ± 0.9	0.232
NPI dysphoria	18	1.2 ± 2.5	9	0.4 ± 1.3	0.298
Number of patients with high/normal CSF p-tau value	18	2:16	9	1:8	0.721^b^
FP-CIT SPECT SBR-average	16	5.5 ± 1.2	9	5.5 ± 1.2	0.934
Number of patients with/without DESH	18	16:2	9	7:2	0.582^b^

iNPH, Idiopathic normal pressure hydrocephalus; APA, Apathy; NPI, Neuropsychiatric inventory; AS, Apathy scale; MMSE, Mini mental state examination; FAB; Frontal assessment battery; ACE, Addenbrooke’s cognitive examination; WAIS, Wechsler adult intelligence scale; DSST, Digit symbol substitution Test; RBMT, Rivermead behavioral memory test; IR, immediate recall; DR, delayed recall; EMC, everyday memory checklist;  TUG, Timed up and go test; WT, walking test; GSSR, Gait status scale-revised; OABSS, Overactive bladder symptoms score; IPSS, International prostate symptom score; iNPHGS, iNPH grading scale; mRS, modified Rankin scale; CSF, cerebrospinal fluid; p-tau, Phosphorylated tau; FP-CIT, [^123^I]-2beta-carbometoxy-3beta-(4-iodophenyl)-N-(3-fluoropropyl) nortropane; SPECT, single-photon emission computed tomography; SBR, specific binding ratio; DESH, disproportionately enlarged subarachnoid space hydrocephalus.

Data represent mean ± SD.

If CSF p-tau value was judged to be normal, when it was below 50 pg/ml, and abnormal, when it was 50 or over.

*p < 0.05.

^a^Mann–Whitney U test.

^b^Fisher’s exact test.

### Comparison of rGMV between the two groups

**Figure 1:**
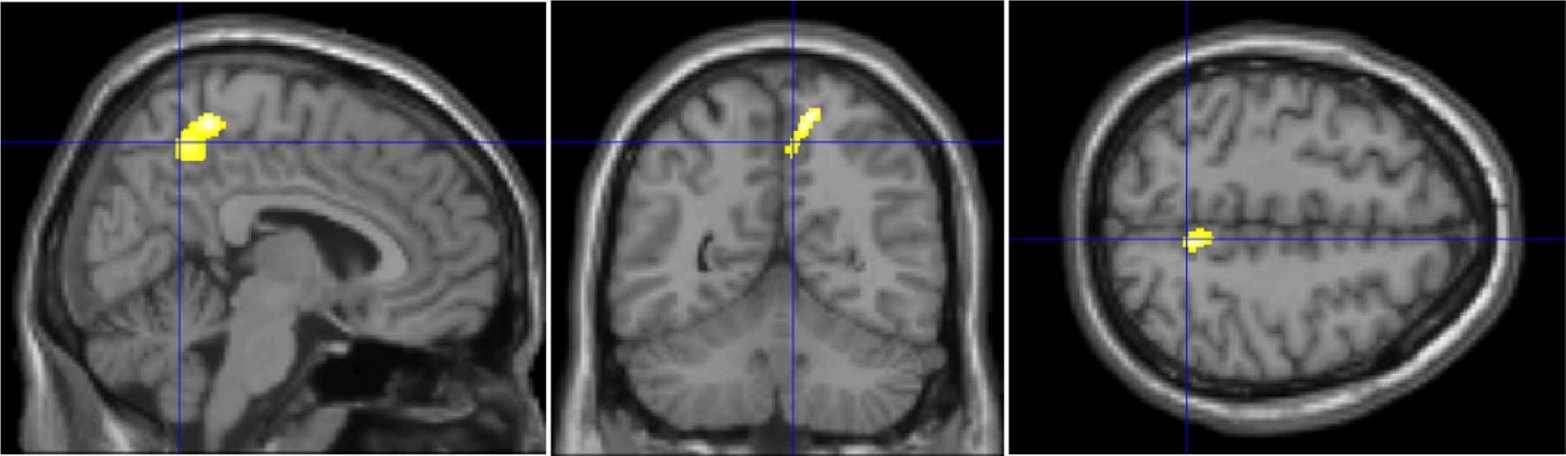
Comparison of rGMV between the two groups.

The rGMV of the right precuneus was significantly larger in the iNPH + APA group than in the iNPH – APA group [*p*(FWE) < 0.05]. There were no brain regions in which the rGMV was significantly smaller in the iNPH + APA group than in the iNPH-APA group (Table 2, Fig. 1).

**Table 2:** MNI coordinates of the brain regions with significant differences in rGMV between the two groups.

GMV	Brain region	Tarairach coordinates					Cluster—level	
		Side	x	y	z	t	p _FWE-corr_	Cluster Level (Voxels)
iNPH + APA > iNPH-APA	precuneus	rt.	14	–54	63	5.46	0.023	503

GMV, Gray matter volume; iNPH, Idiopathic normal pressure hydrocephalus; APA, Apathy; FEW-corr, Family wise error-corrected; rt., right.

### Comparison of rCBF between the two groups

There were no brain regions with significant differences in rCBF between the iNPH + APA and iNPH – APA groups.

## Discussion

To the best of our knowledge, this is the first study to investigate the neurological basis of apathy in patients diagnosed with probable iNPH using SPM12 to examine both rGMV and rCBF. The analysis showed that the rGMV of the right precuneus was larger in patients with iNPH with apathy than in those without apathy. In iNPH, a larger rGMV of high-convexity areas indicates greater grey matter compression^12^. However, there were no brain regions with significant differences in the rCBF between the two groups. These results suggested that the dysfunction in the precuneus might be involved in apathy in iNPH and that the grey matter compression might be more important than the abnormality of the rCBF for the apathy of iNPH. In addition, this is the first report to suggest the association between grey matter compression and clinical symptoms in iNPH; therefore, this study might facilitate the elucidation of the pathogenesis of the symptoms of iNPH. This study might also indicate that the VBM is a useful method of assessing grey matter compression in iNPH patients.

The iNPH patients with apathy had significantly worse GSSR scores and tended to have higher TUG, WT, and EMC scores and lower MMSE scores than those without apathy in this study, indicating presence of more severe gait and cognitive disturbances in those with apathy. The findings of this study are consistent with those of previous studies^8,9^. In addition, iNPHGS urinary and NPI dysphoria scores also tended to be worse in iNPH patients with apathy than in those without apathy in this study. To exclude these effects based on the above results, our SPM/VBM analysis included gait, cognition, and urination scores of iNPHGS, MMSE score, and NPI dysphoria score, in addition to age and sex, as covariates. In this analysis, which was adjusted for the effects of various factors, was possible because the specialists, including neuropsychiatrists, neurologists, urologists, radiologists, and neurosurgeons, cooperate well and the comprehensive evaluations for iNPH were performed at Kochi University Medical Hospital.

The results of this study showed no significant differences between the two groups in the number of patients with CSF phosphorylated tau levels > 50 and SBR values of FP-CIT SPECT. AD-related pathology is highly prevalent^41,42^ and striatal dopaminergic dysfunction was observed^43^ in patients with iNPH. Apathy in patients with neurocognitive disorders might be associated with AD-related pathology and dopaminergic dysfunction of the basal ganglia, in addition to age, sex, depression, and cognitive impairment^44^. However, AD comorbidity and dopaminergic dysfunction of the basal ganglia likely had little influence on the results of this study.

In this study, a relatively large rGMV in the right precuneus was shown to be associated with apathy. In NPH, vascular compliance is lower in a compressed superior sagittal sinus than in a straight sinus that is less compressed; the decreased vascular compliance of the compressed veins has believed to decrease the pulsation of the arteries^45^. Therefore, the right precuneus compression shown in this study may be presumed to have decreased the vascular compliance of the veins and arterial pulsation. Decreased arterial pulsation lowers the extravascular flow of CSF and lymphatic fluid and impairs the fundamental system of eliminating waste products from the interstitial space^46^. Furthermore, it has been suggested that the accumulation of waste products increases the osmotic pressure of spinal and extracellular fluids and that an elevated osmotic pressure may cause neuronal and axonal dehydration and contribute to neuronal and axonal damage and dysfunction in iNPH^47^. The right precuneus, which was shown to have a relatively large rGMV in this study, strong compression can impair the function of its neurons or axons; based on this, we surmised that right precuneus dysfunction was associated with apathy.

The results of this study suggested that the dysfunction in the right precuneus might be involved in apathy in iNPH. Among patients with PD, atrophy of the precuneus and decreased glucose metabolism were reported in those with apathy than in those without apathy, which revealed that precuneus volume and glucose metabolism were negatively correlated with the severity of apathy^48,49^. In addition, a previous study using functional MRI showed that the precuneus, along with the posterior cingulate cortex, is involved in both thinking about one's own intentions and consequent actions and bearing in mind an intention to make an action^50^. Specifically, in the right precuneus, a previous study using VBM on healthy participants reported that gray matter density positively correlated with the availability of manifold information, imagination, and problem solutions^51^. A functional MRI study of adolescents also reported that adolescents with higher-risk sexual behavior demonstrated greater activation in the right precuneus to social rewards^52^, suggesting that the right precuneus is involved in responses to social rewards. These findings of previous studies collectively led us to surmise that right precuneus dysfunction may impair the processes to generate behavior and presentation of apathy.

This study found no association between rCBF and apathy, although a previous study of 56 patients with iNPH demonstrated a relationship between apathy in iNPH and decreased CBF in the right caudate nucleus^24^. The methodological difference may have influenced the difference in the results; this study used whole-brain analysis, whereas the previous study used ROI analysis. A previous study on 97 patients with iNPH using whole-brain analysis showed an association between urinary disturbance and decreased rCBF in the right frontal lobe^53^. Therefore, no significant differences in rCBF between the two groups in this study might be due to the small sample size.

The small sample size of this study may bias the characteristics of the subjects and raise the concern that its results may not be those of a study conducted on common iNPH patients. First, in this study, we used strict diagnostic criteria for probable iNPH per the Japanese guidelines for the management of iNPH [Mori second version^25^], which resulted in a smaller sample size. Japanese guidelines for the management of iNPH emphasize DESH. DESH is a characteristic finding in iNPH that is useful differentiating it from other type of dementia^11^ and identifying iNPH patients who are more likely to benefit from shunt surgery^54^. In this study, the proportion of participants with DESH was high (85%), and we believe that we selected iNPH patients with a high degree of homogeneity. Second, we compared apathy between patients in the present study and those in two previous studies. A previous study evaluated psychiatric symptoms in 63 patients with iNPH^3^, and another study evaluated apathy in 56 patients with iNPH^24^. The prevalence of apathy was 66.7% in this study, 70.3% in Kito’s study, and 73.2% in Kanemoto’s study. The mean NPI-apathy score in iNPH patients with apathy was 4.8 in this study and 5.5 in Kanemoto’s study (no available data for Kito’s study). The prevalence and degree of apathy were a little less and milder in this study; however, apathy in iNPH patients in this study is similar to that in patients in previous studies. Third, we compared the triad of symptoms and characteristics between iNPH patients in this study and those in previous studies, including a multicenter prospective cohort study of 100 iNPH patients conducted in Japan^11^. In this study, the mean scores of iNPHGS gait, cognition, and urination were 2.0, 2.3, and 1.8, respectively. However, it is remarkable, that the mean of each of the three iNPHGS scores in Kito’s study (gait:2.0, cognition: 2.3, urination: 1.8) is the same. In SINPHONI, mean scores of iNPHGS gait, cognition, and urination were 2, 2, and 2, respectively (no available data for Kanemoto’s study). The mean MMSE scores were 21.8 in this study, 20.6 in Kito’s study, 22.2 in Kanemoto’s study, and 23.0 in SINPHONI. The mean age was 78.0 years in this study, 74.9 years in Kito’s study, 76.6 years in Kanemoto’s study, and 74.5 years in SINPHONI. The percentage of males was 59% in this study, 59% in Kito’s study, 55% in Kanemoto’s study, and 58% in SINPHONI. The mean Evans indices, which indicate the degree of enlargement of the lateral ventricles, were 0.34 in this study and 0.36 in SINPHONI. Therefore, the participants of this study can be considered common iNPH patients, although their mean age was a little higher than that of patients in previous studies.

This study had several limitations other than a small sample size. First, our data were not compared to those of normal controls; thus, we cannot conclude that the gray matter volume in the precuneus is larger than that of elderly normal control subjects. Second, apathy was examined in terms of gray matter volume and CBF, leaving open the possibility that other factors, such as white matter damage due to compression, may be related to apathy. Therefore, the results of this study should be reinforced in the future, and further studies using larger sample sizes and normal control groups are required to better understand the neural basis of apathy in iNPH. In addition, it is necessary to standardize research methods to allow for comparisons between different studies.

## Conclusions

Patients with iNPH manifesting apathy were found to have large rGMV of the right precuneus, suggesting that the intense compression of the right precuneus is associated with apathy in patients with iNPH. In addition, this study shows that VBM is useful for indicating the degree of brain compression. Therefore, a longitudinal follow-up of patients who have DESH but do not have remarkable triad symptoms^55^ using VBM might reveal the pathogenesis of symptoms of iNPH.

## Data Availability

The materials and datasets are available from the corresponding author upon reasonable request.

## Abbreviations

iNPH: Idiopathic normal pressure hydrocephalus
MRI: Magnetic resonance imaging
SPECT: Single photon emission computed tomography
VBM: Voxel based morphometry
ROI: Region of interest
DARTEL: Diffeomorphic anatomical registration through exponentiated lie algebra
